# Integrated Analysis of Morphological, Physiological, Anatomical and Molecular Responses of Cassava Seedlings to Different Light Qualities

**DOI:** 10.3390/ijms241814224

**Published:** 2023-09-18

**Authors:** Qin Zhou, Ruimei Li, Alisdair R. Fernie, Yannian Che, Zhongping Ding, Yuan Yao, Jiao Liu, Yajie Wang, Xinwen Hu, Jianchun Guo

**Affiliations:** 1 School of Life Sciences, Hainan University, Haikou 570228, China; 21210710000025@hainanu.edu.cn (Q.Z.); liruimei@itbb.org.cn (R.L.); 22110710000011@hainanu.edu.cn (Y.C.); 21220951310010@hainanu.edu.cn (Z.D.); 2 Key Laboratory of Biology and Genetic Resources of Tropical Crops, Institute of Tropical Bioscience and Biotechnology, Chinese Academy of Tropical Agricultural Sciences, Haikou 571101, China; yaoyuan@itbb.org.cn (Y.Y.); liujiao@itbb.org.cn (J.L.); wangyajie@itbb.org.cn (Y.W.); 3 Key Laboratory for Biology and Genetic Resources of Tropical Crops of Hainan Province, Hainan Institute for Tropical Agricultural Resources, Haikou 571101, China; 4 Max-Planck-Institute of Molecular Plant Physiology, Am Muhlenberg 1, 14476 Potsdam, Germany; fernie@mpimp-golm.mpg.de

**Keywords:** light quality, cassava, plant growth, antioxidant, carbohydrate content, gene expression, chlorophyll, tissue culture, in vitro

## Abstract

Light quality is highly important for growth control of in vitro plant cultures. Here, we investigated the effect of blue light (BL), red light (RL) and combined red and blue light (RBL) on in vitro cassava growth. Our results indicate that RL facilitated radial elongation of cassava and increased stomatal conductance as well as glucose, sucrose, fructose and starch content in leaves and cellulose content in the stem. It also enhanced SOD and POD activities but decreased the stomatal density and chlorophyll and carotenoid content in leaves. In addition, RL leads to shorter palisade cells, denser chloroplasts and more starch granules. These phenotypic changes were inverted following BL treatment. The expression levels of photosynthesis-related genes *MeLHCA1*, *MeLHCA3*, *MePSB27-2*, *MePSBY*, *MePETE1* and *MePNSL2* in leaves were at their lowest following RL treatment, while the expression levels of *MePSB27-2*, *MePSBY*, *MePETE1* and *MePNSL2* were at their highest after BL treatment. The phenotypic changes after RBL treatment were between the values observed for the RL and BL treatments alone. Moreover, the responses of SC8 and SC9 cassava varieties to light quality were largely conserved. As such, we believe that the results of this study lay the foundation for controlling the in vitro growth of cassava seedlings by light quality.

## 1. Introduction

Cassava (*Manihot esculenta* Crantz) is ranked the sixth most important crop in the world and is planted globally in tropical and subtropical areas. Cassava tuberous root contains large reserves of starch, which comprises approximately 85% of the dry weight [1,2]. As such, cassava is a stable crop that feeds almost one-tenth of the global population. Cassava can additionally be used as a biomass crop. For now, the most common method of cassava propagation is taking stem cuttings. However, this approach is somewhat prone to introducing diseases that threaten cassava production [3,4]. Tissue culture technology is, therefore, increasingly seen as an effective alternative method by which to rapidly obtain a large number of virus-free plantlets [5]. In addition, cassava grown in the field is likely to be attacked by viruses, bacteria and pests, as well as damaged by cold and salt stress. Moreover, the postharvest quality of cassava decreases rapidly [2,6,7,8,9]. To overcome these limitations, gene manipulation strategies are increasingly being taken in order to generate new varieties. Given that the in vitro culture of seedlings is a prerequisite for producing transgenic lines, it follows that tissue culture technology plays an important role in cassava variety improvement.

Light is a crucial factor for plant growth, as it not only provides energy and power for photosynthesis to facilitate the biosynthesis of organic compounds but also regulates morphologic changes and metabolism in the plant life cycle [10,11]. Light is perceived in plants through various photoreceptors, such as phytochrome, cryptochrome and phototropins, to control photomorphogenesis [12]. Light quality/spectrum refers to the color or wavelength of light. The light spectrum that directly affects plant photosynthesis ranges from 400 to 700 nm. The peak absorbance of chlorophyll is at 430 to 450 nm and 640 to 660 nm [13]. Thus, blue light, which ranges from 420 to 500 nm, and red light, which ranges from 620–700 nm, are both critically important for plant growth. Early studies evidenced the contribution of light quality to plant growth, reproduction, defense and metabolism [14,15,16]. Red and blue lights are the main subjects of study due to their prominent roles in the regulation of plant life activities [12]. For example, red and blue lights affect plant height, biomass, stem diameter, and bud numbers in opposing directions [17,18,19]. Red and blue lights are the main light types that regulate stomatal movement, but this is subject to significant species-specific differences [20,21]. Red and blue lights also regulate the accumulation of chlorophyll a, carotenoid and flavonoid production [22]. Light quality is a critical environmental factor in tissue culture. Recently, reports have studied the impact of various light spectra on the growth of plants in vitro in many species, including pitaya [17], potato [18], *Cinchona officinalis* [19], *Leucojum aestivum* [22], *Swertia chirayita* [23], *Pyrus communis* [24], Valencia orange [25] and banana [26]. However, to date, no such study has been conducted on in vitro-grown cassava plants.

For this reason, the current study aimed to reveal the differences in the plant growth, physiology, anatomical structure, and photosynthesis-related gene expression of cassava cultured in vitro under different light quality regimes. These experiments additionally compared whether there were differences in response to light quality between two different varieties of cassava.

## 2. Results

### 2.1. Effect of Light Quality on In Vitro Growth of Cassava

**Figure 1:**
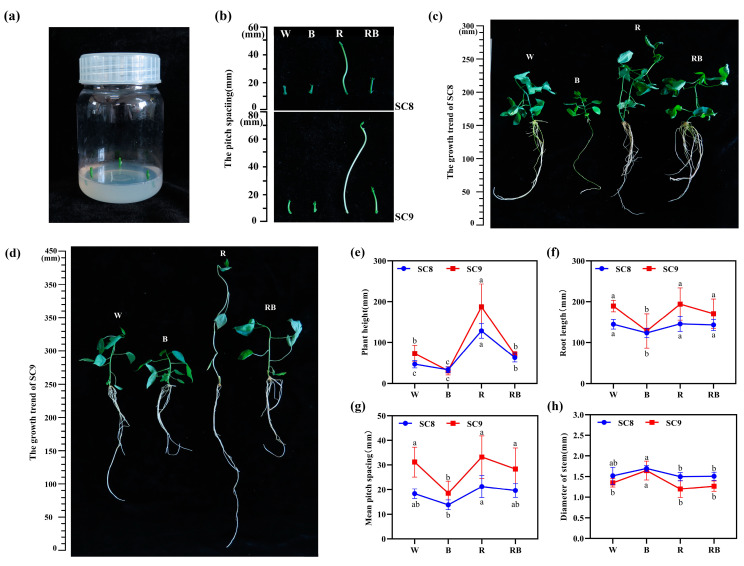
Effects of different light qualities on in vitro cassava growth. (**a**) Stem segment of cassava single-axillary bud before light treatment. (**b**) The third internode of cassava under different light. (**c**) Phenotype of SC8 cassava under different light. (**d**) Phenotype of SC9 cassava under different light. Morphological parameters, including plant height (**e**), root length (**f**), mean pitch spacing (**g**) and diameter of stem (**h**) in cassava under different light qualities. W, white light; B, blue light; R, red light; RB, red:blue (8:1) light. All data were represented by mean and standard deviation, and Duncan’s multivariate range test was used, with different letters indicating significant differences (*p* < 0.05; N = 6).

In order to understand the effects of different light qualities on the in vitro growth of cassava, stem segments with a single bud from the cultivars SC8 and SC9 were exposed to white light (WL, control, 450–460 nm), blue light (BL, 400–520 nm), red light (RL, 610–720 nm) and mixed red and blue light (RBL, R:B = 8:1), respectively, in a 16/8 h (light/dark) photoperiod at 28 °C for 40 days (Figure 1a). Our results revealed that different light qualities significantly affected cassava growth in vitro (Figure 1). BL significantly inhibited the radial growth of cassava seedlings; by contrast, RL significantly promoted the radial growth of cassava seedlings, while the effect of RBL on plant height was similar to that of the control treatment WL (Figure 1b,c). The measurements of plant height, root length, average pitch distance and stem diameter revealed that plant height, root length and average pitch distance all decreased, while stem diameter increased under BL treatment. However, the above indices showed inverse change under RL treatment, for which plant height, root length and average pitch distance were at least mildly increased, and stem diameter was at least mildly decreased (Figure 1e–h). The values of plant height, root length, average pitch spacing and stem diameter of tissue-cultured cassava seedlings under RBL treatment were all intermediate between BL and RL and were close to those of the control WL treatment (Figure 1e–h). In addition, the phenotypic trends of SC8 and SC9 cassava varieties were highly similar under different light quality treatments (Figure 1b–h).

### 2.2. Effect of Light Quality on Stomatal Density and Stomatal Aperture of Cassava

**Figure 2:**
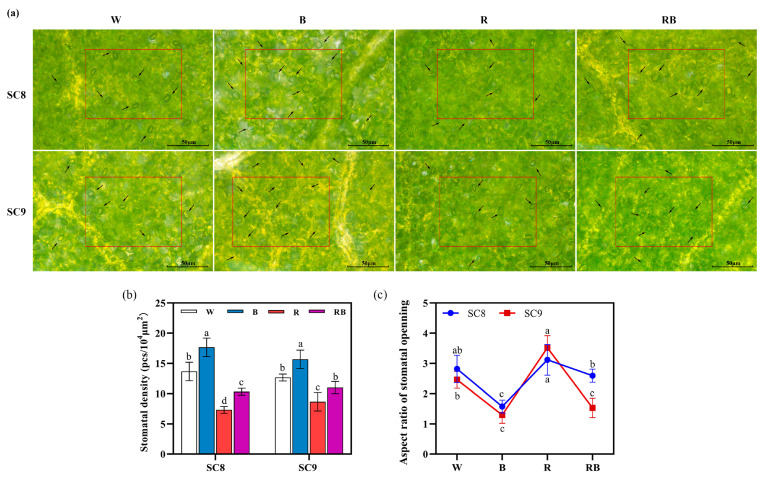
Influence of light quality on cassava stomatal density and aperture. (**a**) Leaf stomata observation after different light quality treatments. The red box indicates scope for stomatal density measurement, which is 10^4^ μm^2^. The arrows point out the stomata. (**b**) Influence of light quality on cassava stomatal density. (**c**) Influence of light quality on cassava stomatal aperture. W, white light; B, blue light; R, red light; RB, red:blue (8:1) light. All data were represented by mean and standard deviation, and Duncan’s multivariate range test was used, with different letters indicating significant differences (*p* < 0.05; N = 6).

To know whether light quality affects the distribution and opening and closing of stomata in cassava leaves, we next observed stomata in cassava leaves cultured under different light conditions under the electron microscope (Figure 2a). Our results revealed that different light qualities significantly affected the stomatal density of leaves. The stomatal density was the highest under BL treatment and lowest under RL treatment, while under RBL treatment, it was higher than RL treatment but lower than BL and WL treatments (Figure 2b). The stomatal aperture was also, to some extent, affected by light quality. BL treatment significantly reduced the stomatal aperture, and RL enhanced the stomatal aperture, while the RBL treatment exhibited a stomatal aperture that was intermediate between BL and RL levels and near control levels (Figure 2c). Furthermore, the patterns of change in stomatal density and aperture under different light qualities were highly consistent between the SC8 and SC9 genotypes (Figure 2b,c).

### 2.3. Effect of Light Quality on Photosynthetic Pigment Content

**Figure 3:**
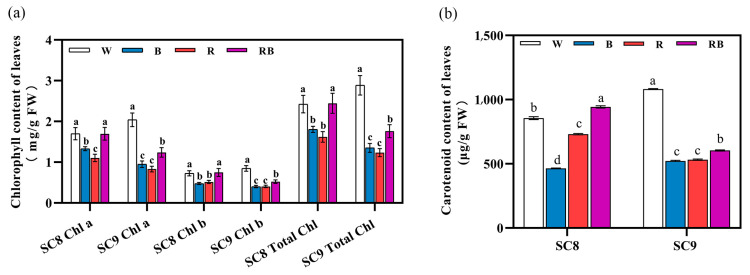
Effects of light quality on cassava photosynthetic pigment content. (**a**) The chlorophyll a, chlorophyll b and total chlorophyll content in SC8 and SC9 leaves after different light treatments. (**b**) The carotenoid content in SC8 and SC9 leaves after different light treatments. W, white light; B, blue light; R, red light; RB, red:blue (8:1) light. All data were represented by mean and standard deviation, and Duncan’s multivariate range test was used, with different letters indicating significant differences (*p* < 0.05; N = 6).

Photosynthetic pigments can absorb and transfer light energy, which is critical in the primary photochemical reaction in photosynthesis and vital for plant growth. In order to clarify whether light quality affects the accumulation of photosynthetic pigments, the content of chlorophyll a, chlorophyll b, total chlorophyll and carotenoid was measured (Figure 3). As shown in Figure 3, compared with the control, the content of chlorophyll a, chlorophyll b, total chlorophyll and carotenoid decreased when monochromatic BL or RL was provided. The application of RBL resulted in pigment levels that were higher than those following BL or RL treatment but still lower than the control, with the exception of the content of carotenoid, which was higher under RBL than the control in SC8. This disparity notwithstanding, other changes in photosynthetic pigment levels were highly similar between the two cassava genotypes.

### 2.4. Effects of Different Light Qualities on Antioxidant Enzyme Activities, Carbohydrate and Soluble Protein Contents

**Figure 4:**
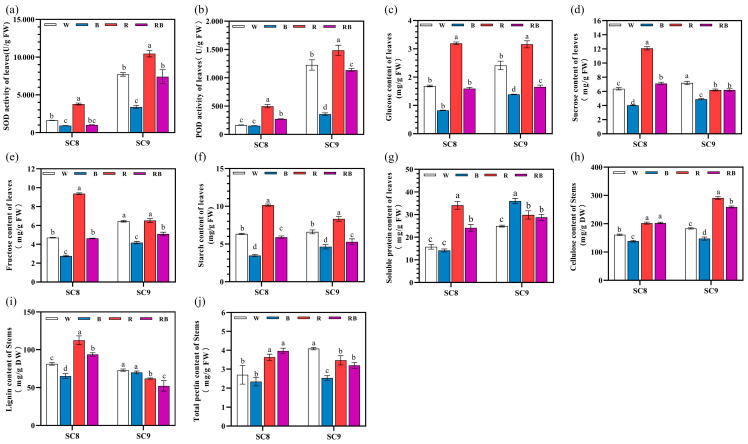
Effects of different light qualities on antioxidant enzyme activities, carbohydrate and soluble protein content in cassava. (**a**) SOD activity of leaves. (**b**) POD activity of leaves. (**c**) Glucose content of leaves. (**d**) Sucrose content of leaves. (**e**) Starch content of leaves. (**f**) Fructose content of leaves. (**g**) Soluble protein content of leaves. (**h**) cellulose content of stems. (**i**) lignin content of stems. (**j**) Total pectin content of stems. W, white light; B, blue light; R, red light; RB, red:blue (8:1) light. All data were represented by mean and standard deviation, and Duncan’s multivariate range test was used, with different letters indicating significant differences (*p* < 0.05; N = 3).

Under BL treatment, the SOD and POD activities were both significantly decreased, while under RL treatment, both SOD and POD activities were significantly increased compared to WL treatment. After RBL treatment, the SOD and POD activities were intermediate between BL and RL treatments, being close in value to those of WL treatment. Moreover, the pattern of change in SOD and POD activities under different light was similar between the two genotypes (Figure 4). BL treatment reduced the content of glucose, sucrose, fructose, and starch in both genotypes. RL treatment significantly enhanced the glucose and starch content in both genotypes and enhanced the sucrose and fructose content in SC8 but decreased or did not affect the sucrose and fructose content in SC9. RBL maintained glucose, sucrose, fructose and starch content intermediate between BL and RL treatments in both SC8 and SC9 (Figure 4). Light quality also affected the content of cell wall components in the stem. BL treatment decreased cellulose, lignin and total pectin content in both genotypes. RL and RBL treatments increased the cellulose in both SC8 and SC9; however, they increased the lignin and pectin content in SC8 but decreased those in SC9 (Figure 4). In SC8, BL decreased but RL and RBL increased the soluble protein content. However, in SC9, BL, RL and RBL all resulted in an increased soluble protein content in comparison to the control (Figure 4).

### 2.5. Effects of Different Light Qualities on Cell Structure of Cassava

**Figure 5:**
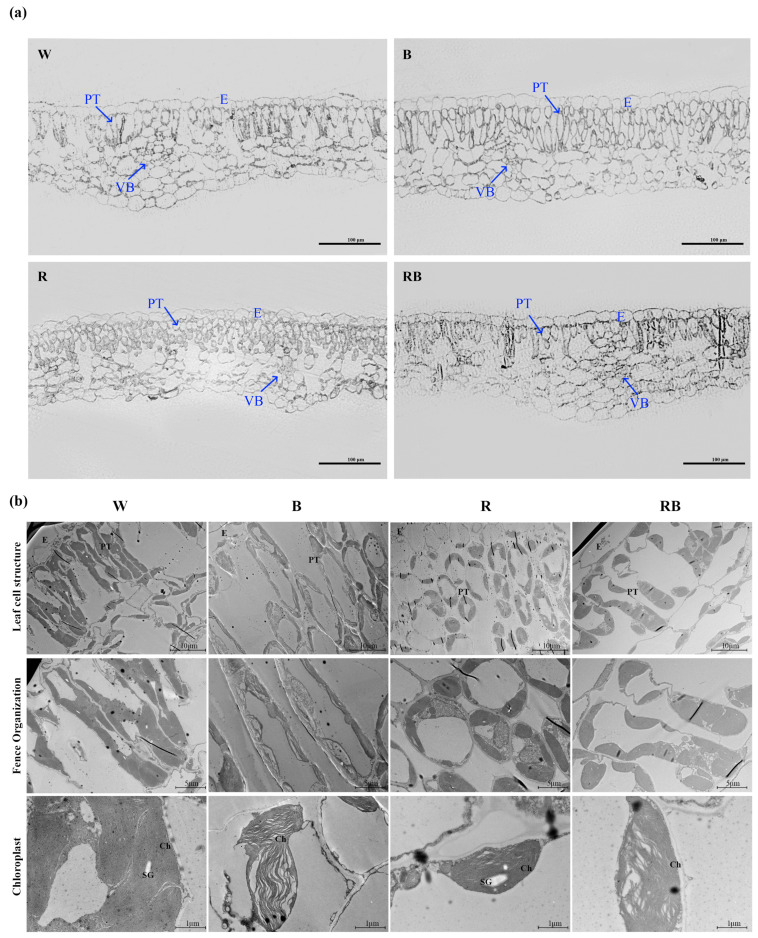
Effects of light quality on cell structure of cassava. (**a**) Semi-thin sections of cassava leaves under different light. (**b**) Chloroplast changes under different light qualities. PT, palisade tissue; E, epidermal; VB, vascular bundle; Ch, chloroplast; SG, starch granule; W, white light; B, blue light; R, red light; RB, red:blue (8:1) light.

To reveal whether light quality affects cell structure in cassava, we made semi-thin sections of cassava leaves treated with different light qualities. Compared to WL-treated leaf epidermal cells, monochromic BL-treated plants had larger leaf epidermal cells, while monochromic RL-treated plants had irregularly shaped and unevenly arranged epidermal cells. Compared to control plants treated by WL, which had palisade tissue cells with an inverted triangle shape, the monochromic BL-treated leaf palisade tissue cells displayed an elongated spindle shape or shortened oval shape, whereas monochromic RL-treated leaves displayed smaller and round-shaped palisade tissue cells. Under monochromic BL treatment, the spongy tissue cells varied in size, and those cells under RL treatment were mid-sized. By contrast, epidermal cells, palisade tissue cells, spongy tissue cells, and even the vascular bundle cells under RBL treatment showed similar characteristics to the control (Figure 5a). Chloroplasts in palisade tissue cells were next observed by transmission electron microscopy. The results showed that the internal stroma lamellae structure of the chloroplasts was tight under the control WL treatment. The stromal lamellae structure of the chloroplasts was still tightly arranged following monochromic RL treatment but loose and disordered after BL and RBL treatment (Figure 5b).

### 2.6. Effects of Different Light Qualities on the Expression of Photosynthesis-Related Genes

**Figure 6:**
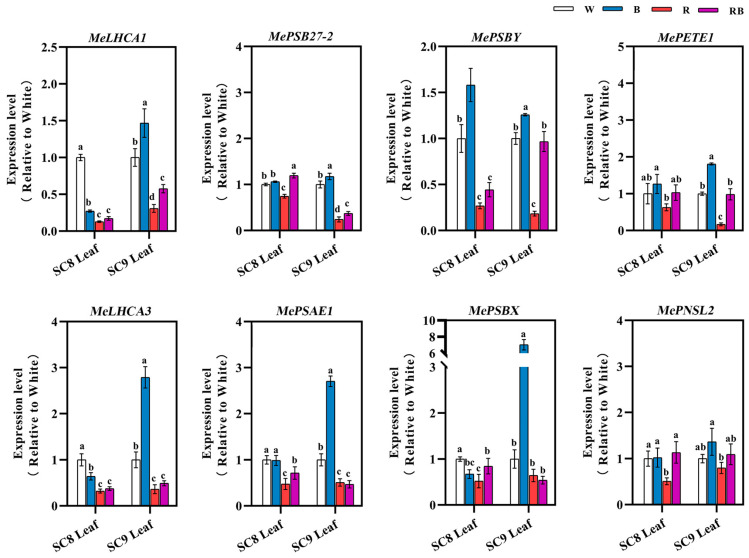
Expression of photosynthesis-related genes in leaves of SC8 and SC9 cassava after different light quality treatments. All data were represented by mean and standard deviation, and Duncan’s multivariate range test was used, with different letters indicating significant differences (*p* < 0.05; N = 3).

To characterize whether the expression of photosynthesis-related genes was affected by different light treatments, two light-harvesting genes (*MeLHCA1* and *MeLHCA3*), one photosystem Ⅰ protein gene (*MePSAE1*), four photosystem Ⅱ protein genes (*MePSB27-2*, *MePSBY*, *MePSBX* and *MePNSL2*) and one plastocyanin gene (*PETE*1), which is the long-range electron carrier between photosystem Ⅰ and photosystem Ⅱ, were selected for assessing their expression levels by qRT-PCR following different light treatments. The results are presented in Figure 6. The expression of *MeLHCA1* and *MeLHCA3* was significantly induced in leaves of SC9 following BL treatment but repressed in SC8 after BL treatment and repressed in both genotypes following RL treatment. The expression of four photosystem Ⅱ protein genes (*MePSB27-2*, *MePSBY*, *MePSBX* and *MePNSL2*) was inhibited by RL treatment in both genotypes. The expression of *MePSB27-2*, *MePSBY* and *MePNSL2* was induced by BL treatment in both genotypes. However, the expression of *MePSBX* was induced by BL and depressed by RL in SC9 but depressed in SC8 following both BL and RL treatments. *MePETE1* was induced by BL and reduced by RL in both genotypes, while the expression level following RBL treatment was intermediate between the BL and RL treatments and similar to that of the control. Expression of the *MePSAE1* gene was reduced by BL and RL treatment in SC8 but induced by BL and reduced by RL in SC9. In general, following RBL treatment, the expression level of these genes was intermediate between the BL and RL treatments in both SC8 and SC9, with the exception of *MePASE1*, *MePSBX* and *MePNSL2*.

## 3. Discussion

Light quality is one of the most critical factors regulating the growth behavior of plants. In our study, the stem elongation of two cassava varieties was inhibited by monochromatic BL but elevated by monochromatic RL compared to WL, whereas these changes were counteracted by the simultaneous irradiation of BL and RL (RBL), wherein the stem length of cassava seedlings was intermediate to the values of the monochromatic light-treated samples and close to that under WL (Figure 1). This result is consistent with findings in other plants, such as in tomato [27,28,29], soybean [30] and cucumber [31], where negative responses of plant radial growth to BL were reported. Moreover, in tomato [30], cucumber [31] and *Mesona chinensis* [32], positive responses of plant radial growth to RL were found. However, there are also reports indicating the positive role of BL on plant growth; for instance, BL enhanced the shoot length and bud numbers of in vitro-cultured *Cinchona officinalis* [19] and promoted hypocotyl elongation of arugula [33]. Similarly, the highest-level BL treatment showed the highest plant height, leaf number, total leaf area, and leaf biomass in *Hydrocotyle bonariensis* [34], with similar results apparent in eggplant treated by BL [35]. Additionally, some studies have found that RL inhibits the elongation of plant height; for example, in eggplant, plant height was inhibited by RL, but the inhibition was rescued by BL [35]. These results suggest that different species of plants respond differently to light quality, with further experimental studies being necessary to understand how particular plants respond to light quality.

Stomata are critical structures in the epidermis that control gas exchange and water loss. As such, stomatal aperture is a major factor affecting photosynthesis, respiration and transpiration of plants [36]. It was found that blue light increased the stomatal density but decreased the stomatal opening of cassava seedlings, while red light decreased the stomatal density and increased the stomatal opening (Figure 2). Previous studies indicate that light quality is one of the regulatory factors controlling stomatal movement, in which RL and BL are the two main light types [21]. RL-induced stomatal movement has been considered to be dependent on photosynthesis, while BL-induced stomatal movement is considered to be photosynthesis independent [21]. Furthermore, a previous study indicated that BL-induced stomatal movement has significant species-specific differences [20]. Under low-light conditions, most plants reduce their stomatal density [37]. BL treatment in tomatoes reduced the stomatal conductance and increased the intercellular CO_2_ concentration, which indicates that BL can reduce the photosynthetic gas exchange of tomato leaves. Whether and—if yes—how the stomatal changes in cassava leaves under different light qualities impact photosynthesis and photosynthetic gas exchange requires further detailed study.

Carbohydrates are important for plant growth, and the influence of light quality on carbohydrate levels has also been reported. In our study, the content of glucose, fructose, sucrose and starch was highest in cassava leaves after RL treatment, while it was lowest after BL treatment (Figure 4). Similar results were reported in lettuce, where the soluble sugar content was increased under RL [38], and in kiwifruit, where RL increased sucrose and starch content [39]. We speculate, on the basis of our results, that the increase in carbohydrates by RL is beneficial to the radial growth of cassava plants.

Antioxidant enzyme activities have previously been reported to be influenced by different light quality treatments [40]. It seems that the activities of antioxidant enzymes regulated by light quality are different in different plant species. In the present study, we observed that the SOD and POD activities in cassava leaves were significantly elevated by RL treatment but inhibited by BL. In contrast, SOD activity was increased following BL treatment in *Rubus hongnoensis* [40], pea [41] and wheat [42].

Both chlorophyll and carotenoids are important pigments for photosynthesis in green plants; therefore, their accumulation is crucial for plant growth. In the current study, we find that the use of monochromatic RL or BL has a negative effect on the accumulation of chlorophyll a, chlorophyll b, total chlorophyll and carotenoid content in cassava leaves. However, exposure to RBL mitigated the negative effects on chlorophyll accumulation (Figure 3). In lettuce, the chlorophyll content was higher following BL and RL treatment. Similarly, the chlorophyll content was higher in cabbage grown under BL, whilst, in kiwifruit, BL had a positive effect on the induction and accumulation of chlorophyll content [39]. Moreover, in apples, BL was found to promote the accumulation of chlorophyll a and b, whereas RL was found to restrict chlorophyll accumulation [43]. In other studies, the carotenoid content in pak choi exposed to pure WL was higher than that following exposure to only BL or RL [44], whereas exposure to BL increased the carotenoid levels, and exposure to RL reduced them [45]. From these findings, it can be seen that the accumulation of photosynthetic pigments requires a variety of light qualities.

In addition to the photosynthetic pigment content, light quality also affects the cellular structure of plants. We found that the palisade cells were obviously elongated in shape following exposure to monochromic BL but shortened following exposure to monochromic RL (Figure 5). Spherical-shaped palisade cells were previously found in the leaves of shade-grown plants [46]. It thus seems that monochromatic RL culture has a similar effect on cassava seedlings as shade, whilst monochromatic BL promotes contrary changes. In addition, the cassava leaves had dense chloroplasts after monochromic RL treatment but incompact chloroplasts after BL treatment (Figure 5). These results are in direct contrast with those of a previous study of apples, which displayed dense chloroplasts following BL treatment [43]. This further highlights the species-specificity of plant responses to light. Following exposure to RL, the spongy and palisade cells were neatly arranged in potato plantlets, while the chloroplasts were well developed under BL [47]. It has additionally been demonstrated that RL can effectively promote the accumulation of starch particles in chloroplasts [48]. However, the accumulation of starch particles may be unfavorable to the photosynthetic activity of plant leaves [49]. In the present study, we observed that the accumulation of starch particles in chloroplasts after RL treatment was higher than that after other treatments. As mentioned above, further studies will be important to determine whether this affects the photosynthetic activity of cassava leaves.

The influence of different spectra on the development of and physiological changes in plants was originally derived from changes in the expression of light-dependent genes [50]. In our work, we showed that the expression levels of several photosynthesis-related genes, including photosystem II protein D1 (PSB), light-harvesting complexes (LHC) and plastocyanin genes, were higher under BL irradiation than those under RL irradiation (Figure 6). The higher expression of PSB genes under BL than under RL was also detected in Scots pine seedlings [50]. Previous studies found that *LHC* gene expression directly influences chlorophyll accumulation [51,52,53]. In our study, the total chlorophyll content in leaves has a similar change trend as the gene expression levels of *MeLHCA1* and *MeLHCA3* after BL or RL treatment. One of the most important events occurring in plant photosynthesis is the capture of light, a process mediated by antenna proteins such as light-harvesting complexes (LHC) [54]. Therefore, it is speculated that light quality may regulate the growth of cassava by altering the expression of the *MeLHCA* gene, regulating the accumulation of chlorophyll and, thereby, affecting the efficiency of photosynthesis. Reverse genetic experiments to test this hypothesis are an obvious priority for future research.

## 4. Materials and Methods

### 4.1. Plant Materials and Light Treatments

Four-week-old sterile tissue-culture seedlings of SC8 and SC9 were used to produce stem sections with a single axillary bud. The stem sections were planted in Murashige and Skoog (MS) solid medium and cultured at 28 °C under 16 h light/8 h dark in different light qualities, including monochromic blue light (400~520 nm), monochromic red light (610~720 nm) and a mix of blue and red light (R:B = 8:1); white light (450–460 nm) was used as the control. Each treatment had 10 bottles, each containing three stem segments.

### 4.2. Observation of Growth and Stomatal Characteristics of Cassava

After 40 days of different light treatments, the cassava seedlings were taken out from the tissue-culture bottles to measure phenotypic data, including plant height, root length, mean internodal distances and stem diameter. Six biological replicates were used for each treatment. For the stomatal studies, the second fully splayed blade of each treatment was used. An inverted microscope (Olympus DP74, Olympus Inc., Tokyo, Japan) was used to observe and measure the stomatal density and take photos. The cellSens Dimentsion version 4.2 (Olympus Inc., Tokyo, Japan) software was used to measure the aperture of stomata.

### 4.3. Determination of Photosynthetic Pigment Content

The roots, stems and leaves of 40-day-treated cassava seedlings were harvested and ground into powder in liquid nitrogen. Approximately 0.1 g samples were used to extract a suspension using an ethanol and acetone mixture (1:4 *v*/*v*). The absorbance of the suspension was measured at 663 nm, 645 nm and 450 nm using an ultraviolet spectrophotometer (UV BlueStar A, Beijing, China) to calculate the chlorophyll a, chlorophyll b and carotenoid concentrations. Three biological replicates were used for each treatment.

### 4.4. Determination of SOD and POD Activities

To measure the superoxide dismutase (SOD) activity in cassava leaves after different light treatments, the total SOD activity assay kit (Suzhou Comin Biotechnology Co., Ltd., Suzhou, China) was used for extraction, and absorption of the suspension was measured at 450 nm using an ultraviolet spectrophotometer (UV BlueStar A, Beijing, China). For the POD activity determination, the peroxidase (POD) activity assay kit (Suzhou Comin Biotechnology Co., Ltd., Suzhou, China) was used, and the absorption of the suspension was measured at 420 nm using an ultraviolet spectrophotometer (UV BlueStar A, Beijing, China).

### 4.5. Determination of Soluble Protein and Carbohydrate Content

Cassava leaves treated under different light qualities were collected. To measure the content of soluble protein, a kit (Suzhou Comin Biotechnology Co., Ltd., Suzhou, China) was used for the extraction, and then the absorbance of the suspension was measured at 562 nm. For glucose, sucrose, starch and fructose, the respective kits from Suzhou Comin Biotechnology (China) were used according to the instruction manuals. The absorbances of the suspensions were measured at 505 nm, 480 nm, 562 nm and 620 nm, respectively.

Cassava stem samples were used to determine the content of total pectin, cellulose and lignin. For total pectin content measurement, the total pectin assay kit (Suzhou Comin Biotechnology Co., Ltd., Suzhou, China) was used, and the absorbance of the suspension at 530 nm was detected. For cellulose content measurement, the cellulose content kit (Suzhou Comin Biotechnology Co., Ltd., Suzhou, China) was used for extraction, and the absorption of the suspension at 620 nm was detected. For lignin content measurement, the lignin content kit (Suzhou Comin Biotechnology Co., Ltd., Suzhou, China) was used, and the absorption of the suspension at 280 nm was detected.

### 4.6. Microstructure Measurements

Cassava leaves cultured under different light qualities were soaked in 2.5% glutaraldehyde • 0.1 M phosphate buffer (pH 7.4), fully permeated in vacuo for 30 min and fixed at 4 °C for 2–4 h. The fixed tissue was then rinsed with 0.1 M phosphate buffer (pH 7.4) three times, for 15 min each time; the solution was then refixed with 1% osmic acid • 0.1 M phosphate buffer (pH 7.4) for 2 h, and again subsequently rinsed with 0.1 M phosphate buffer (pH 7.4) three times, for 15 min each time. Then, 30~100% ethanol was used for step-by-step dehydration. The mixture was then permeated with acetone:embedding agent at a ratio of 1:1 for 1 h. Subsequently, acetone and embedding agent were used at a ratio of 1:3, and the mixture was permeated overnight. Then, the tissue was transferred to pure resin for embedding. Subsequently, polymerization was carried out at 37 °C (12 h)–45 °C (48 h)–60 °C (48 h). After trimming the embedded block, semi-thin slices and ultra-thin slices were prepared. The semi-thin sections were stained with toluidine blue solution, and cell structure was observed using an electron microscope and photographed. The ultra-thin sections were double-stained with uranium lead (uranium dioxyacetate and lead citrate for 15 min each), and the microstructure was observed using a transmission electron microscope and photographed.

### 4.7. Expression Analysis of Photosynthesis-Related Genes

The total RNA of roots, stems and leaves of cassava was extracted by using a plant total RNA isolation kit (Foregene, Chengdu, China) and purified by using MonScript RTIII super mix with dsDNase (MonScript, China). Then, the total RNA was reverse-transcribed into cDNA. The qRT-PCR reaction system was performed according to the instructions of the SYBR^®^ Premix Ex TaqTM II reagent (Takara, Kusatsu, Japan). The ABI 7900 HT fast real time PCR system was used to detect the fluorescence threshold and cycle threshold (Ct) value of each gene after different light treatments. The relative expression levels of each gene were calculated using the 2^−ΔΔCT^ method. The cassava tubulin gene was used as a housekeeping gene. All the primers of the genes are listed in Table S1. In addition, three biological replicates were used in this experiment.

### 4.8. Statistical Analysis

All data collected were statistically analyzed using Statistics 20.0 (IBM Corporation, Armonk, NY, USA). The one-way ANOVA and Duncan’s multiple range tests were combined to test whether the effects of each treatment were significant.

## Data Availability

The datasets generated during and/or analyzed during the current study are available from the corresponding author on reasonable request.

## Supplementary Materials

The supporting information can be downloaded at: https://www.mdpi.com/article/10.3390/ijms241814224/s1.
